# Aged-related Function Disorder of Liver is Reversed after Exposing to Young Milieu *via* Conversion of Hepatocyte Ploidy

**DOI:** 10.14336/AD.2020.1227

**Published:** 2021-08-01

**Authors:** Qinggui Liu, Fei Chen, Tao Yang, Jing Su, Shaohua Song, Zhi-Ren Fu, Yao Li, Yi-Ping Hu, Min-Jun Wang

**Affiliations:** ^1^Department of Cell Biology, Center for stem cell and Medicine, Second Military Medical University (Naval Medical University), Shanghai, China; ^2^Changzheng Hospital, Second Military Medical University (Naval Medical University), Shanghai, China; ^3^State Key Laboratory of Genetic Engineering, Fudan University, Shanghai, China.

**Keywords:** rejuvenation, hepatocyte polyploidy, liver regeneration, liver steatosis, young milieu

## Abstract

Previous study showed that senescent hepatocytes from aged liver could be rejuvenated after repopulated in the young recipient liver. The proliferative capacity of hepatocytes was restored with the senescence reversal. However, it is unknown whether metabolic and homeostatic function of aged liver, as well as age-dependent liver steatosis could be rejuvenated or alleviated. Here, we found that senescent hepatocytes from aged liver were rejuvenated after exposing to young blood. An autonomous proliferation of senescent hepatocytes which resulting in ploidy reversal might be the underlying mechanism of senescent reversal. After performing 2/3 partial hepatectomy (2/3PHx) in young blood exposed old liver, delayed DNA synthesis of senescent hepatocytes was rescued and the number of BrdU positive hepatocytes was restored from 4.39±2.30% to 17.85±3.21%, similarly to that in the young mice at 36 hours post 2/3PHx. Moreover, Cyclin A2 and Cyclin E1 overexpression of hepatocytes in aged liver facilitating the G1/S phase transition was contributed to enhance liver regeneration. Furthermore, lipid droplet spread widely in the elderly human liver and old mouse liver, but this aged-associated liver steatosis was alleviated as senescence reversal. Collectively, our study provides new thoughts for effectively preventing age-related liver diseases.

Liver aging promotes functional deficiencies of metabolic and homeostatic regulation and provokes a gradual decline in regenerative properties, resulting in the progressive loss of the liver's capacity to self-sustain [1]. Evidence revealed that aging was the most important risk factor for a multitude of liver diseases, such as metabolic disorders with accumulation of lipid droplets, regenerative deficiency, and liver fibrosis [2-6]. Most of liver functions are carried out chiefly by hepatocytes, which account for 70% of all liver cells. However, hepatocytes become senescent with the ageing process [7]. Senescent hepatocytes show striking variation in gene expression, including changes of known cell-cycle inhibitors or activators, accompanied by growth arrest on failing to initiate DNA replication [8, 9]. Senescent hepatocytes can also be identified by DNA-damage foci, which leading to repressing genes expression that encode proteins to regulate metabolism [10, 11]. Senescent hepatocytes accumulated in liver is thought to be associated with deteriorating liver functions and is a predominant risk factor for liver disorders. Therefore, it is urgently required to understand mechanisms of reversal in senescent hepatocytes. This might improve the function of aged liver, attenuate age-associated liver diseases, and even promote the quality of life for elderly individuals.

Our previous study demonstrated that senescent hepatocytes from aged liver could be rejuvenated after engrafting into young recipient, implying that young microenvironment of recipient liver contributed to restoring the proliferative potential of senescent hepatocytes [12]. While young donor hepatocytes were transplanted into young or old recipient livers, the liver repopulation in old recipients was significantly lower than that in young recipients. However, increased systemic level of Growth hormone (GH) in old recipient liver can enhance its liver repopulation after cell transplantation, further supporting the positive role of young systemic environment in reversal of cellular senescence [13]. It is also reported that young plasma could prevent liver aging and reduce the sensitivity of aged livers to ischemia reperfusion injury through ameliorating aging-induced suppression in hepatic autophagy [14, 15].

Parabiosis model has been applied to study the systemic environment impacting on organ function and recovery from damage. Heterochronic parabiosis is a process that joins young mice with old mice, allowing for a shared circulatory system. Based on previous research applying parabiosis model, improved recovery in age-related diseases has been observed in myogenesis, neurogenesis, memory and cognitive abilities, cardiac hypertrophy, and gastrointestinal-related disease [16-22]. To date, no study has examined the effect of a youthful systemic environment on liver homeostatic sustains and metabolic functions.

Based on above, we wondered whether changes in systemic blood and secreted proteins might be crucial for reversal of liver regenerative impairment and metabolic dysfunction such as liver steatosis. Here, through performing heterochronic parabiosis, we found that senescent hepatocytes in old liver were reversed when exposed to young blood. An autonomous proliferation of senescent hepatocytes resulted in ploidy reversal might be a major contributing factor to rejuvenation. Importantly, the capacity of liver regeneration for self-sustain after damage was recovered and aged-associated steatosis was alleviated after exposing to young milieu.

## MATERIALS AND METHODS

### Animals and Human samples

Male C57BL/6 mice were obtained from Shanghai Sippe-Bk Lab Animal Co. Ltd. The parabiotic partners including the young (8 weeks) and the aged (22-24 months) were maintained under a temperature-controlled room with 12-h light/dark cycles and unlimited access to food and water. mTmG C57BL/6 mice (Jackson, Stock No. 007676) was used to identify the circulatory system share, in which the ACTB promoter allows strong and persistent expression of red fluorescence in cellular membrane from all tissue and cell types. Only 3 pairs of mTmG and wildtype C57BL/6 mice were performed parabiosis experiment. The other parabiotic partners were all wildtype C57BL/6 background mice. All animal breeding, care and experimentation procedures were in accordance with animal ethics committee of the Chinese PLA general hospital and military medical college on animal protection.

Human liver tissues were provided by Changzheng Hospital, Naval Medical University (Shanghai, China), which were from normal liver tissues adjacent to hepatic hemangioma after a surgical resection. Patients’ characteristics and clinical features are listed in Supplementary Table 1. Each patient gave written informed consent. Experiments were approved by Ethical Committee on Ethics of Biomedicine Research, Naval Medical University.

### Parabiosis model

Surgical procedures that followed the methods of previous researchers [23] were applied to connect two mice and eatablish a shared blood circulation. Animals were anesthetized, shaved and sterilized. Skin incisions were made starting at 0.5 cm above the elbow all the way to below the knee joint in the shaved sides. Then the joining was defined by connecting the knee and skin joints respectively. The injection of anti-inflammatory Carprofen (5mg/kg) and the analgesic Buprenorphine (0.1mg/kg) by subcutaneously was given as directed for pain every 24 and 12 hours, respectively, for 48 hours during recovery. And treat mice with Sulfamethoxazole/Trimethoprim (2 mg Sulfa/mL+ 0.4 mg Trim/mL) oral suspension in their water bottle for 7 days to prevent bacterial infections. Monitor animals for signs of pain and distress. Peripheral blood was collected from the mandibular venous plexus of each mouse for flow-cytometric analysis to confirm blood chimerism in 2 weeks later. After 5 weeks, the livers were harvested to analyze the rejuvenation of senescent hepatocytes, the proliferative capacity of hepatocytes, and the metabolic functions. Two thirds partial hepatectomy (PHx) surgeries were performed 8 weeks post parabiosis surgery. Isochronic parabiosis were the sham group, included Iso-O were stand for old-to-old parabiosis (22-24 months), and Iso-Y were stand for young-to-young parabiosis (10 weeks). Heterochronic parabiosis were young mouse jointed with old mouse. Het-Y represents the young mouse, and its partner was name Het-O.

### Liver perfusion, primary hepatocyte culture and analysis of cell proliferation

Mice were anesthetized and then the liver was perfused in situ with collagenase D solution (Roche, Indianapolis, IN) as previously described [24]. Isolated Hepatocytes were seeded on 24-, or 6-well plates coated with mouse tail collagen in Hepatic Cell Culture Medium (Invitrogen, Carlsbad, CA). After 6 hours, non-adherent cells were removed by washing the wells with phosphate buffered saline. For the proliferation assay, cells were incubated for 12 hours with BrdU labeling regent (100uM, Sigma-Aldrich, St. Louis, MO). And then cells were fixed with a 1:1 mixture of ice-cold methanol:glacial acetic acid. Following treating with 2N HCl for 30 min at room temperature, cells were incubated with primary and second antibody as with the IHC protocols.

### Analysis and isolation of hepatocyte ploidy by Flow Cytometry

The detection of hepatocyte ploidy populations by flow cytometry performed as previously described [12]. In brief, hepatocytes isolated from parabiotic mice were fixed in ice-cold 75% (vol/vol) ethanol (Sinoreagent, Shanghai, CHN), then incubated with 50μg/mL propidium iodide (Sigma-Aldrich, St. Louis, MO) in phosphate-buffered saline supplemented with 1μg/mL (wt/vol) RNAse A (Sigma-Aldrich, St. Louis, MO). DNA content of a minimum of 30,000 nuclei per liver sample was analyzed with a FACS-Calibur flow cytometer (BD Biosctoences, San Jose, CA).

For isolation of hepatocyte ploidy populations, the hepatocytes were incubated with 15μg/mL Hoechst 33342 for 30 min at 37°C. Cells were sorted with InFlux flow cytometer using a 150μm nozzle. Dead cells were excluded on the basis of 5μg/ml propidium iodide. Populations of different ploidy cells were identified with parameter of DNA content using a 355nm laser and 425-440nm bandpass filter. Sorted hepatocytes were collected in DMEM containing 50% fetal bovine serum. Only the highly purified populations were used for further experiments.

### Partial hepatectomy model and BrdU intake assay

Two thirds partial hepatectomy (PHx) surgeries were performed as previously described [24]. The parabiotic mice were anesthetized and both the median and left lobes were removed in two mice respectively. Bromodeoxyuridine (BrdU) (Sigma-Aldrich, St Louis, MO) was injected intraperitoneally with 100 mg/kg (body weight) in PBS at 2 hours before killing the animals. The livers were dissected, and parts of livers were fixed in 4% formalin and stored in room temperature. The remaining liver were embedded in O.C.T. compound (Tissue-Tek) or frozen in liquid nitrogen and stored in -80?.

### Staining for senescence-associated β-galactosidase (SA-β-Gal) and cell size measurement

As published procedures described [25, 26], Senescence Detection Kit (Abcam, ab65351) was used to analysis the SA-β-Gal activity in frozen liver section. In brief, the frozen sections were fixed with fixative solution for 10 min at room temperature, washed with PBS and were incubated in fresh SA-β-Gal staining (a solution buffer at pH 6.0) for 12 hours at 37°C. Washed after incubation and counterstained with Nuclear Fast Red (Vector Laboratories, Burlingame, CA). Sections were dehydrated and mounted with 30% glycerol medium. And 15 random fields were imaged per sections. Senescent hepatocytes were counted as a percentage of all hepatocytes per field. For cell size measurement, fluorescent images from β-catenin-stained tissue sections were downloaded onto a computer in which cell boundaries were outlined in red pixels. The number and area of hepatocytes were quantified by Image Pro Plus software.

### Telomerase activity and telomere length assay

As previously described [12], quantification of telomerase activity was performed with a telomerase PCR ELISA kit (Roche, Indianapolis, IN). Telomere length was determined by q-PCR using the modified Cawthon’s method. Genomic DNA was extracted from isolated mouse hepatocytes using the DNeasy Blood & Tissue Kit (QIAGEN, Valencia CA) according to the manufacturer’s instructions.

### Immunohistochemistry (IHC) and immunecytchemistry (ICC)

Immunohistochemical staining for H_2_A histone family, member X (γ-H_2_AX, Abcam, ab81299), BrdU (Abcam, ab6326), Ki67 (Abcam, ab15580), Cyclin A2 (Abcam, ab181591), Cyclin E1 (Abcam, ab52189) and Cyclin D1 (Abcam, ab134175) were performed on 4% paraformaldehyde-fixed liver sections. 5μm-thick slices were incubated with primary antibodies at 4? overnight followed by biotinylated secondary antibody and the avidin/biotin horseradish peroxidase system (Vectastain DAB Kit; Vector Laboratories, Burlingame, CA). The nuclei were counterstained with hematoxylin (Sigma-Aldrich, St. Louis, MO) and the sections were covered in neutral balsam (Solarbio, Beijing, CHN). Immuno-fluorescent staining for β-catenin (Sigma-Aldrich, MAB2081) and PHH3 (Roche, 760-4591) were detected on paraffin-embedded liver section. Slides were incubated with primary antibodies at 4 °C overnight and then with secondary antibodies conjugated with fluorescent dye at 37°C for 30 min.

Immunocytochemical staining for BrdU (Abcam, ab6326) and Ki67(Abcam, ab15580), cells were fixed, permeabilized and blocked, then incubated with primary antibody, followed by fluorescence-tagged secondary antibodies. Nuclei were counterstained with Hoechst 33258 (Sigma-Aldrich, St. Louis, MO). Images were acquired with a 50i Nikon fluorescence microscope (Nikon, Melville, NY).

### Oil Red O and Nile red staining

Oil Red O (Sigma-Aldrich, St. Louis, MO) working solution, Nile Red solution (Sigma-Aldrich, St. Louis, MO) and frozen OCT-embedded liver slides were prepared according to previously described [4]. Briefly, Oil Red O working solution was the mixed liquid of 3: 2 of stock solution and water. The working solution filtered by 0.45-μm filter was applied on 7-μm OCT- embedded liver section for 20 min. 2μL Nile Red solution (150μg/mL in acetone) was added into 1mL 80% glycerol. Frozen liver sections were air dried for 5 min and fixed for 10 min with 4% paraformaldehyde dissolved in PBS. Following rehydration in PBS, DAPI solution (Sigma-Aldrich, St. Louis, MO) was applied on fixed liver section for 5min. And the Nile Red solution was directly added to slide, mounted on a glass microscope slide and covered with a cover slip. Images were taken immediately after mounting. Image J software was used to quantify lipid droplets by measuring areas of red pixels.

### Protein isolation and western blot

Western blot analysis was performed as described previously [12]. 50μg liver total proteins were homogenized with RIPA lysis buffer (Boston Bio Products) supplemented with protease and phosphatase inhibitors (Roche, Indianapolis, IN) according to manufacturer’s instruction. The protein was separated by acrylamide gels, and transferred to a PVDF membrane (Millipore, Temecula, CA). Membranes were blocked and incubated with primary antibodies against the following proteins at 4 °C overnight: β-Actin (Thermo Fisher, PA1-16889); P53(Abcam, ab23); P21(Abcam, Ab7960); P16(Abcam, ab186034); Cyclin A2(Abcam, ab181591); Cyclin D1(Abcam, ab134175); Cyclin E1(Santa Cruz, sc37710). After being washed, the membranes were incubated with HRP conjugated antibodies. Then ECL reagents were used for chemiluminescence detection. Protein bands were visualized by chemiluminescent substrate (Thermo Scientific, Waltham, MA), and densitometric analysis was performed in Image pro plus.

### Serum biochemistry analysis

All serum samples were collected and centrifuged at 1000 g for 10 minutes and stored at -80°C before analysis. The analysis of ALT (Biovision, K752-100), AST (Abnova, KA4187), TG (Biovision, K614-100), LDL-c (Biovision, K585-100) and cholesterol (Biovision, K587-100) in serum sample were performed according to the manufacturer’s instructions.

### Statistical analysis

All experiments were performed at least three times. Data were presented as mean ± standard deviation (S.D.). Statistical significance was determined with a two-tailed unpaired Student’s *t*- test in two groups, whereas one-way ANOVA was performed in analysis of more than two groups. P values <0.05 were considered as statistically significant. The data analysis was performed using GraphPad Prism 5.0c for Mac (Graph Pad Software).

## RESULTS

### Senescent hepatocytes in the aged liver could be reversed to youthful state when exposed to young blood.

**Figure 1.**
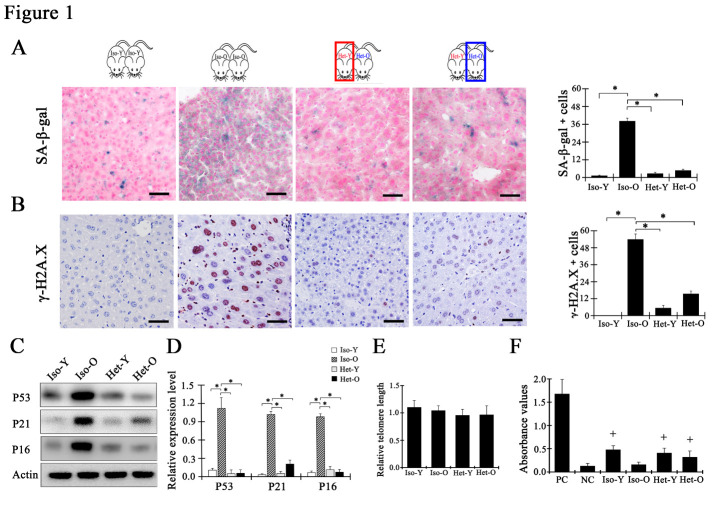
The senescent hepatocyte in aged liver was rejuvenated when exposed to young blood. (A) Senescence-associated β-galactosidase (SA-β-Gal) staining results of liver tissues from Iso-Y, Iso-O, Het-Y and Het-O. Blue precipitates in cytoplasm are SA-β-Gal positive hepatocytes. The value represented as mean ± SD. *p<0.05, the bar=50μm, n=5 pairs. (B) Representative images showing γ-H2A.X staining of the liver sections from respective group. Brown precipitates in nucleus are γ-H2A.X positive cells. Graph representing the percentage of γ-H2A.X positive cells in the liver from indicated group. The bar=50μm, the data shown as mean ± SD. *p<0.05, n=5 pairs. (C) Western blotting analysis the expression of senescent biomarker P53, P21 and P16 in proteins extracts from one animal of indicated group. (D) The expression of P53, P21and P16 was analyzed by western blotting. Protein expression in Iso-O mouse was taken as the baseline and considered equal to 1. Values are represented as mean ± SD. *p<0.05, n=5 pairs. (E) The telomere length of hepatocytes was detected by a quantitative polymerase chain reaction (PCR) assay from Iso-Y, Iso-O, Het-Y and Het-O. (F) Telomerase activity was detected by TeloTAGGG telomerase PCR enzyme-linked immunosorbent assay (ELISA (Roche). “+” was regarded as telomerase positive (absorbance [ΔA] is higher than 0.2 A_450nm_-A_690nm_ units). PC, positive control; NC, negative control. All data are shown as mean ± SD. *p<0.05, n=5 pairs for each group. Iso-Y: the young mouse in isochronic parabiosis between young mice. Iso-O: the aged mice in isochronic parabiosis between aged mice. Het-Y: the young mice in heterochronic parabiosis between young and aged mice, the mice were pointed out by red box. Het-O: the aged mice in heterochronic parabiosis between young and aged mice, the mice were pointed out by blue box.

Our previous study demonstrates that the prevalence of senescent hepatocytes in the liver increases with physiological aging, and these senescent hepatocytes were characterized by high-level senescence-associated β-galactosidase (SA-β-Gal) activity, genomic instability and up-regulation of cell cycle inhibitory protein [27]. To investigate the contribution of young systemic milieu to reverse the senescent hepatocytes in aged liver, we first built a shared circulatory system: young-to-old pairs (Het-Y and Het-O) were the experimental heterochronic parabiosis, two animals of the same aged including young-to-young (Iso-Y) and old-to-old (Iso-O) were the control isochronic pairs. In 2 weeks after parabiosis surgeries, old mice successfully shared half of the young blood and the young ones received the half of old blood (Supplementary Fig. 1). Credibly, the number of senescent hepatocytes with high SA-β-Gal activity was obviously decreased in the liver harvested from the Het-O than that from the Iso-O mice (Het-O: 4.84±0.71% vs Iso-O: 38.23±1.86%) after 5 weeks of shared circulation (Fig. 1A). γ-H2A.X, a maker of persistent DNA damage response, is another hallmark of senescent cells. As shown in Fig. 1B, the number of γ-H2A.X positive hepatocytes in liver from Iso-O was about 54%, while there were few positive cells in liver from Iso-Y. The number of γ-H2A.X-positived hepatocytes was decreased to 15% in Het-O, and it was only slightly increase in Het-Y (Fig. 1B). Western blot analysis indicated that the over-expression levels of P53, P21 and P16 were significantly decreased in the old liver that subjected to young milieu (Fig. 1C, D). With the analysis of telomere length and telomerase activity, we found that compared to young hepatocytes, the telomere length of age hepatocytes remained stable, while the telomerase activity was absent (Fig. 1E, F). But with youthful blood exposure, the telomerase activity of hepatocytes was reactivated in Het-O liver (Fig. 1F). Altogether, these data indicated that the senescent hepatocytes in aged liver were reversed after exposing to the young milieu.

**Figure 2.**
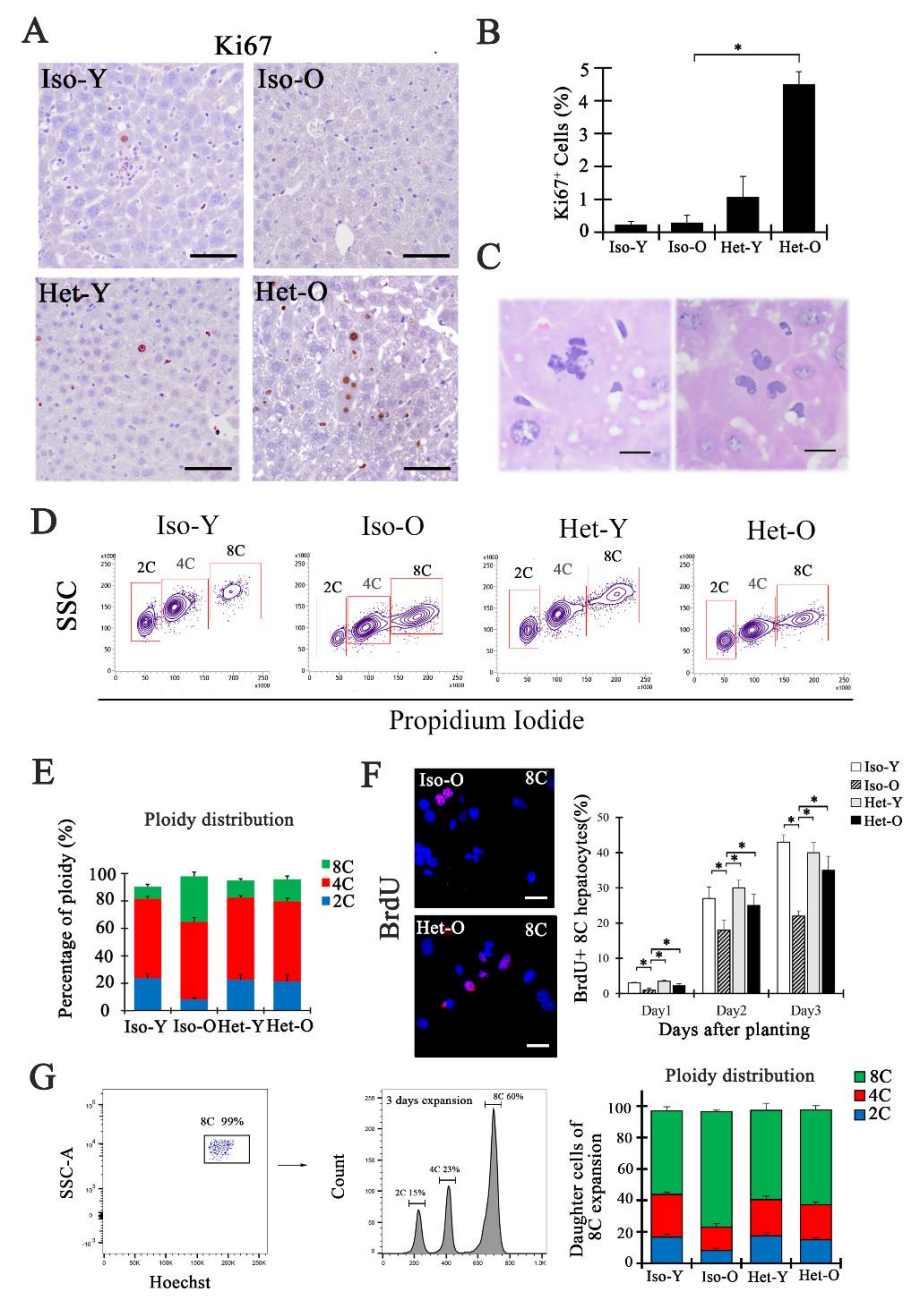
Cell-autonomous proliferation of senescent hepatocytes exposing to young milieu contributes to the polyploidy reduction and hepatocytes rejuvenation. (A) *In situ* immunohistochemical staining of Ki67 in the liver section after parabiosis surgery shows quiescent senescent hepatocytes re-enter into cell cycle. The bar=50μm. (B) Quantitative analysis of Ki67 positive hepatocytes in Iso-Y, Iso-O, Het-Y and Het-O livers. Data are shown as mean ± SD, *p<0.05, n=5 pairs for each group. (C) Hepatocytes dividing *in vivo* formed mitotic figures: multipolar spindles and tripolar division (5weeks after parabiosis). The scale bars =10μm. n=3. (D) Flow cytometry was used to analyze highly pure 2C, 4C and 8C hepatocytes in Iso-Y, Iso-O, Het-Y and Het-O liver at 5 weeks after parabiosis surgery. (E) Graphs indicating the percentage of 2C, 4C, and 8C hepatocytes for each group mice. Data are shown as mean ± SD. *p<0.05, n=5 pairs for each group. (F) BrdU incorporation in cultured 8C hepatocytes from old mice with (down) or without (up) parabiosis at 3 days cultured. The right bar showing the percentage of BrdU-positive 8C hepatocytes from Iso-Y, Iso-O, Het-Y and Het-O mice at day1, day2, and day3 during primary culture. Data are shown as mean ± SD. *p<0.05, the bar=50μm, n=3 pairs for each group(right). (G) Highly pure (99%) 8c hepatocytes were isolated for *in vitro* culture (left). After 3 days of culture, DNA content of cultured hepatocytes was analyzed by FACS (middle). Ploidy distribution ratio in the daughter cells of four parabiosis group (right) was showed as means ± S.D. * p < 0.05, n=3 pairs.

### Cell-autonomous proliferation of senescent hepatocytes contributes to polyploidy reduction and hepatocytes rejuvenation

Given the reactivated telomerase activity, we next sought to examine whether cell proliferation occurred in liver of parabiosis models. Interestingly, we found that quiescent hepatocytes in normal aged liver were re-entered into the cell cycle under exposing to young systemic milieu (Fig. 2A, B). Culture of primary hepatocytes isolated from Het-O and Iso-O mice *in vitro* unveiled that the senescent hepatocytes exposed to the youthful milieu displayed an increased proliferative capacity (Supplementary Fig. 2A-C). More excitingly, a mitotic structure with multipolar spindles and tripolar division was detected *in situ* liver section of Het-O mice, implying that one proliferative polyploidy hepatocytes could generate more than two daughter cells with ploidy reduction (Fig. 2C). The proportion of polyploid hepatocytes in liver increases with age and multipolar cell divisions in polyploid hepatocytes subsequently result in ploidy reduction have been frequently observed [28-32]. To further confirm the polyploidy reversal in the model, we quantified diploid (2C), tetraploid (4C) and octoploid (8C) hepatocytes in the liver from heterochronic and isochronic parabiosis. The results indicated that the proportion of octoploid cells decreased from 33.35±3.83% in Iso-O liver to 15.99± 3.00% in Het-O liver (Fig. 2D, E). Correspondingly, after exposing to young systemic plasma, the percentage of diploid cells in aged liver was obviously increased from 8.11±1.65% to 21.36±5.99%, almost reaching the ratio that seen in young liver (23.77±3.66%). This data suggested that cell-autonomous proliferation of quiescent hepatocytes might be a self-repairment way for reducing the polyploidy and reversing the hepatocytes senescence. Additionally, octoploid hepatocytes were isolated for analysis of their proliferative potential and ploidy distribution. As shown in Fig. 2F, the DNA synthesis level of polyploidy hepatocytes of Het-O mice were significantly increased. And the increasing trend was more pronounced at 3 days, with a 1.5-fold increase in Het-O liver. After 3 days of octoploid hepatocytes culture, about 40% daughter cells were diploid and tetraploid cells that generated with ploidy reduction (Fig. 2G). Meanwhile, diploid hepatocytes from Het-O liver could generate tetraploid and octoploid cells (data not shown). Accompany with polyploid reversal, the number of hepatocytes was increased but hepatocyte areas decreased in aged liver (Supplementary Fig. 2E, F). Together, these results demonstrated that senescent hepatocytes from young blood treated liver performed cell-autonomous proliferation to reduce their polyploidy, which might be the underlying mechanism of hepatocytes rejuvenation and increase in their proliferative capacity.

### The delayed DNA synthesis of PHx-induced aged liver regeneration could be rescued by being exposed to young blood

**Figure 3.**
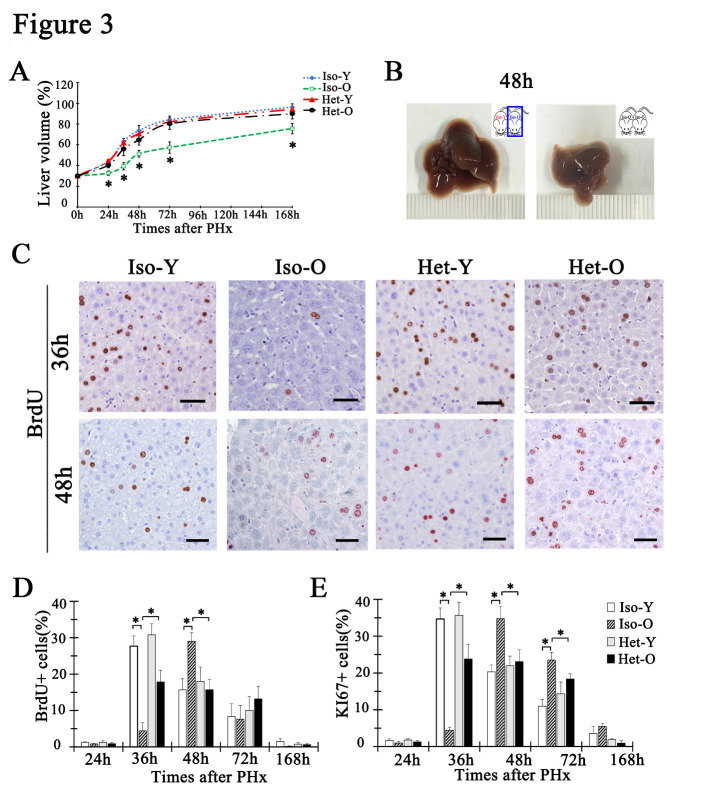
The aged liver regeneration after partial hepatectomy was recovered after young blood exposure. (A) The rate of the liver mass to original liver at 0h, 24h, 36h, 48h, 72h, and 168h post-PHx. One hundred percent represent the remnant liver recover to original weight. Data are shown as mean ± SD. *p<0.05, n=3 pairs. (B) Photographs of representative livers from Het-O and Iso-O 48h post- PHx. ( n=3 pairs) (C) BrdU immunostaining of liver sections at 36h and 48h after PHx in Iso-Y, Iso-O, Het-Y and Het-O mice. The bar=50μm. (D, E) Percentage of BrdU (D) and Ki67-positive (E) hepatocytes were quantified for all groups at the indicated time points after PHx. Nuclei were counted in 10 microscopic vision fields per section. Three sections per mouse were examined. Data are shown as mean ± SD. *p<0.05, n=3 pairs for each group.

The liver has a remarkable capacity to regenerate. Even when 70% of its mass is surgically removed, the remnant hepatocytes would immediately re-enter the cell cycle and generate new hepatocytes to compensate for the lost tissue and functions [33]. The capacity of liver regeneration expressed an age-dependent decline [9, 34-37]. For example, in response to partial hepatectomy, hepatocytes from aged liver delayed entering into cell cycle and that led to a sharply decrease in the number of proliferative cells. In our study, a standard 2/3 partial hepatectomy was performed to evaluate the regenerative function of hepatocytes in parabiosis models. As shown in Fig. 3A, the rate of liver mass recovery in Het-O at 24h, 36h, 48h, 72h and 168h post partial hepatectomy was significantly higher than that in untreated aged liver. And the remnant tissue in aged mouse could achieve a fully liver mass recovery after 2/3 PHx. But it couldn’t recover the lost liver completely in the untreated aged mouse, which was only about 75% of the original liver at 168h post-hepatectomy (Fig. 3A, B). BrdU incorporation analysis was used to detect the S-phase hepatocytes at several timepoints after partial hepatectomy. As shown in Fig. 3C, 36 hours after partial hepatectomy, BrdU positive hepatocytes in young liver were at peak, and then gradually decreased. The number of BrdU positive hepatocytes in Iso-Y was 27.69±2.78% at 36 hours post hepatectomy while there was only 4.39±2.30% in Iso-O. And in the liver of old mice, the peak of BrdU labeling index was delayed to 48 hours, which was 29.04±2.33%. There was no significant difference of BrdU positive hepatocytes at 72h, 168h after partial hepatectomy (Fig. 3C, D). Interestingly, during young blood exposure, the number of BrdU positive hepatocytes in old liver at 36h after 2/3 PHx was sharply increase to 17.85±3.21%. But the BrdU index at 48h post hepatectomy was decreased to 15.71%±2.79%, suggesting that the the peak of BrdU labeling index was restored to 36 hours post hepatectomy. The Ki67 staining data also demonstrated that the peak of hepatocytes proliferation in Het-O liver was shift from 48 hours to 36 hours after 2/3PHx (Fig. 3E). All of above results demonstrated that with the hepatocytes rejuvenation after young blood exposure, the delayed proliferation of aged hepatocytes was improved, and the peak of DNA synthesis index was shift from 48h to 36h post 2/3 PHx.

### G1/S phase arrest of senescent hepatocytes was response for delayed liver regeneration

**Figure 4.**
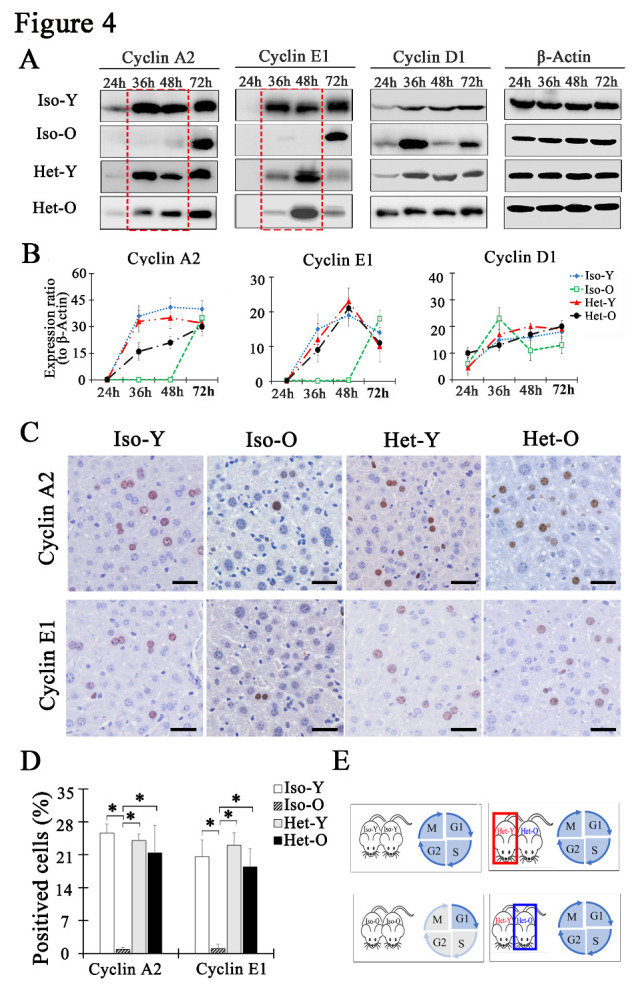
The expression of cell cycle proteins regulated in G1/S check point was restored after PHx. (A) Western blot analysis of Cyclin A2, Cyclin E1 and Cyclin D1 proteins in liver from Iso-Y, Het-Y, Het-O and Iso-O were performed at different times after PHx. β-Actin was used to normalize data. (B) Cyclin A2, Cyclin E1, and Cyclin D1 protein level from the four groups were assayed from western blot. Data are shown as mean ± SD. *p<0.05, n=3 pairs. (C) The representative results of Cyclin A2 and Cyclin E1 by IHC at 36h post-PHx. The bar=50μm. (D) Graph representing the percentage rate of Cyclin A2-positive and Cyclin E1-positive hepatocytes for all groups 36h post-PHx. Data are shown as mean ± SD. *p<0.05, n=3 pairs for each group. (E) A schematic diagram of cell cycle progression post-PHx.

As previously reported, the age-dependent hepatocyte regeneration was impaired at cell cycle arrest of G1/S transition. Thus, we postulated that the successful progression through of G1/S transition might play the major contributor to enhance liver regeneration in the aged. To test this hypothesis, Cyclin E1 (an essential for G1/S transition), Cyclin D1 (a promoter of the late G1 phase) and Cyclin A2 (a controller of the S phase and entry into mitosis) were examined by western blotting. As shown in Fig. 4A, the level of Cyclin A2 and Cyclin E1 was expressed from 36h to 72h after hepatectomy in the young mice. However, their expression levels were absent at 36 and 48h but were only presented at 72 hours in the aged liver post hepatectomy. After subjected to young circulating system, the level of Cyclin A2 and Cyclin E1 from Het-O was increased from 36 to 48h after partial hepatectomy, which was in line with the expression of Het-Y. The expression levels of Cyclin D1 were almost detected at 24, 36, 48, and 72h in all mice, suggested that hepatocytes in young and old mice could enter G1 phase after hepatectomy (Fig. 4A, B). Furthermore, *in situ* immunohistochemical staining showed a marked increase of Cyclin A2 and Cyclin E1 positive hepatocytes in Het-O liver. The number of Cyclin A2 and Cyclin E1 positive hepatocytes was reaching up to 21.39±5.86% and 18.39±3.92% respectively at 36 h post resection while there was only about 1% of both cells in Iso-O liver (Fig. 4C, D). In combination, these data indicated that senescent hepatocytes of aged liver arrested at the G1/S phase was the reason for the delayed liver regeneration. Young milieu induced senescence reversal contributing to ushered hepatocytes through the S-phase after hepatectomy, which was the crucial step for completing liver regeneration (Fig. 4E).

**Figure 5.**
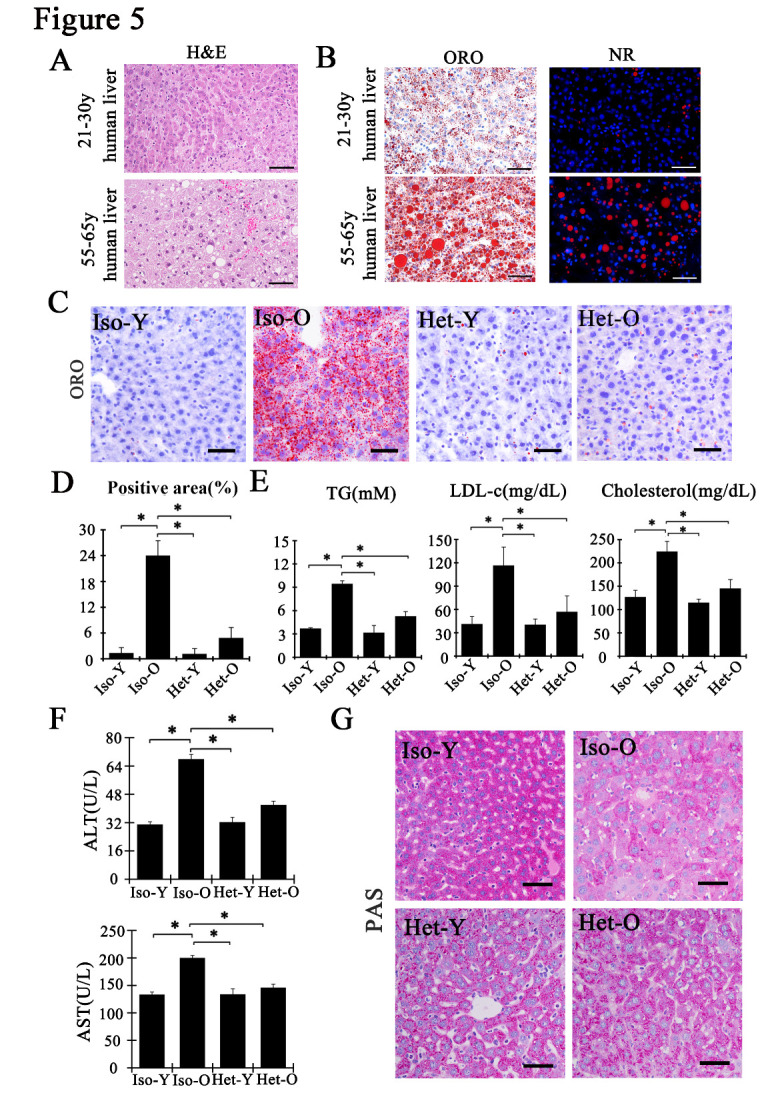
The aged-dependent lipid storage was ameliorated, and liver metabolic function was improved in aged liver. (A) Liver histology of the young and elder human liver subjected by hematoxylin and eosin staining. The scale bars represent 100μm (n=5). (B) Representative histological panels of oil red O staining (red) and Nile red staining in young and old human liver sections. The scale bars represent 100μm (n=5). (C) Representative histological panels of oil red O staining in liver sections from Iso-Y, Iso-O, Het-Y and Het-O mice. The scale bars represent 50μm. (D) Percentage of Oil Red O staining was determined using ImageJ. Data are shown as mean ± SD. *p<0.05, n=5 pairs. (E) Graphs showing the serum levels of TG, LDL-c and cholesterol isolated from Iso-Y, Iso-O, Het-Y and Het-O mice. Data are shown as mean ± SD. *p<0.05, n=5 pairs. (F) Graphs showing serum ALT and AST levels from Iso-Y, Iso-O, Het-Y and Het-O mice. Data are shown as mean ± SD. *p<0.05, n=5 pairs. (G) Representative photomicrograph of liver sections stained with PAS for Iso-Y, Iso-O, Het-Y and Het-O mice. The scale bars represent 50μm.

### Young milieu ameliorates aged-dependent lipid storage and increases liver metabolic function

As introduced previously, the occurrence of steatosis increases with age process [38-40]. Cellular senescence drives age-associated hepatic fat accumulation and steatosis, and removal of senescent hepatocytes can diminish liver steatosis [4]. We evaluated the lipid accumulation in adult and old human liver. As illustrated in Fig. 5A, it was almost not detected any ballooned hepatocytes in adult young human liver, while there was an increase of hepatocellular ballooning degeneration (a character of hepatic steatosis) in aged liver. To achieve a more reliable quantification of lipid storage in aged liver, we applied oil-red O and Nile red staining to test the lipid level. And we found that lipid content was significantly higher in liver from old human as compared to that from the young individuals (Fig. 5B). A similar increase of lipid was observed in aged mice liver (Fig. 5C, D, Supplementary Fig. 3A, B). But after young milieu treatment, there was only one quarter of hepatic fat was observed in Het-O liver compared to that in Iso-O liver (Fig. 5D). Consistent with the decrease of lipid accumulation, triglyceride, low density lipoprotein cholesterol and cholesterol levels of serum from young blood exposed old mice were significantly decreased, suggesting that young milieu protected against fat accumulation in the liver (Fig. 5E). More importantly, the expression of alanine transaminase (ALT) and aspartate transaminase (AST) was reduced in young blood treated age mice (Fig. 5F). Furthermore, the Periodic acid-Schiff staining (PAS, a measure for hepatic glycogen storage) showed a reduction of glycogen content in aged liver. But hepatic intracytoplasmic glycogen content was strongly increased in Het-O mice, suggesting the increased storage of carbohydrates with hepatocyte rejuvenation (Fig. 5G). Token together, we concluded that the aged liver subjected to young milieu could rescue age-dependent liver dysfunction.

## DISCUSSION

Aging, a fundamental biological process accompanied by a general function decline in organs, is the largest risk factor for multitudes of aged-associated chronic diseases. In the liver, the aging phenomena was significantly interacted with the development of chronic liver diseases in a complex way. Aged mice were more susceptible to alcoholic liver injury and hepatocyte senescence was positively correlated with progression of fibrosis [5, 41]. Here we demonstrate that subjected to young milieu, senescent hepatocytes were reverted to a youthful state, and the age-dependent decline in regenerative potential and metabolic dysfunction was improved. Previous studies illustrated that youthful systemic milieu was regarded as positive regulator in the aging process and some age-related diseases. Moreover, the soluble factors from young blood have reported to reverse the activity of somatic stem and some age-related impairments (muscle atrophy, cardiac hypertrophy and acute kidney injury) [21, 42]. TIMP2, a metalloproteinase inhibitor, has been identified as a rejuvenating factor in human umbilical cord plasma and its administration can improve learning and memory in aged mice [43]. Another potential rejuvenating blood factors whose levels decrease with age are growth differentiation factor 11 (GDF11) and hormone oxytocin [42]. Muscle stem dysfunction in aged mice can be cured by direct injections of GDF11 and hormone oxytocin respectively [42]. However, the pro-youthful factor exerting in liver is still unknown and that require new tools to derive insights into the process of hepatic rejuvenation. Encouragingly, recent studies have indicated the human plasma proteomic change between the young and the old individuals, which showing several candidates rejuvenated factors such as GDF15 [44, 45]. It inspires us to apply protein profiling technology or Luminex assay for further elucidating the rejuvenating factors.

Polyploidization, a characteristic feature of hepatocytes, is dynamic. For example, hepatocytes can increase ploidy by failing cytokinesis and produce ploidy-reduced progeny through multipolar mitosis in different contexts. It has been hypothesized that hepatocyte polyploidization may be protection against cellular stress and injury during aging process and age-dependent diseases occurrence [29, 46]. In our study, we found that with the senescence rejuvenation, youthful milieu promoted the quiescent hepatocytes re-entered into cell cycle. And we found several multipolar cell divisions *in situ* liver section of young blood treated old mice, indicating the potential of ploidy reduction. More importantly, the proliferation of cultured octoploid hepatocytes isolated Het-O liver was increased, and these proliferated octoploid cells generated diploid and tetraploid hepatocytes with ploidy reduction. Therefore, we hypothesized that cell-autonomous proliferation of hepatocytes was the driving force for ploidy reduction, which might be the underlying mechanism of hepatocytes rejuvenation. Liver polyploidization has been reported to serve to burden metabolic function. But as shown in our study, the more ploidy hepatocytes in old mice and human, the much weaker function of lipid or glycogen metabolism. In contrast, followed with reversal of senescent hepatocytes and polyploidy reduction, the capacity of liver lipid catabolism was increase and liver steatosis was ameliorated. This led us to hypothesize that hepatocyte polyploidization was compensate for the impaired ability to catabolize fat.

To date, it is well known that the hepatocytes have a powerful capacity to regenerate in response to two-thirds partial hepatectomy. Following removal of two-thirds of rodent liver in the young, it only takes ~7 day before the regeneration was completed [47]. Nevertheless, the remnant liver performed an age-dependent decline following PHx in rodent and human. Here, we described that the aged liver could restore the powerful regenerative capacity after partial hepatectomy when exposed to young systemic milieu. The G1/S phase arrest of senescent hepatocytes in old liver could be abolished with hepatocyte rejuvenation. The peak of hepatocyte proliferation was shift from 48h to 36h after PHx, contributing to the completed liver mass restoration. However, the mechanism of how the senescent hepatocytes passed through G1/S arrest remained to be further studies. Systemic signaling pathways have been implicated in regulating the proliferation of hepatocytes. The decreased level of Growth hormone (GH) as an actual barrier for liver regeneration in aged mouse, GH administration to old-aged mouse dramatically increases proliferative capacity of hepatocytes [48]. By contrast, the fact of age-dependent inhibition of autophagy could be ameliorated by suppressing circulating IGF-1 level [49]. Further study is needed to reveal the underlying mechanism for improving liver regeneration in young circulation.

In summary, this work described senescent hepatocytes became rejuvenated when exposed to young milieu. Most importantly, autonomous proliferation of senescent hepatocytes resulted in ploidy reversal might be a driving force of restoring the age-decreased liver regeneration. Young circulation induced hepatocytes rejuvenation also contributed to alleviate aging-induced hepatic steatosis, liver injury, and deficient glycogen synthesis. These findings suggest that young milieu may provide a potential clue to develop promising therapeutic strategies for age-dependent liver diseases.

## Supplementary Materials

The Supplemenantry data can be found online at: www.aginganddisease.org/EN/10.14336/AD.2020.1226.


