# Supplementary material for: Aged-related Function Disorder of Liver is Reversed after Exposing to Young Milieu via Conversion of Hepatocyte Ploidy
Source: Aging Dis. 2021 Aug 1;12(5):1238–51. doi: 10.14336/AD.2020.1227 (PMC8279529; doi:10.14336/AD.2020.1227)
Supplement: Supplementary file 1 [file AD-12-5-1238-s.pdf]

SUPPLEMENTARY DATA

Aged-related Function Disorder of Liver is Reversed after Exposing to Young Milieu *via* Conversion of Hepatocyte Ploidy

Qinggui Liu[1, #], Fei Chen[1, #], Tao Yang[1], Jing Su[1], Shaohua Song[2], Zhi-Ren Fu[2], Yao Li[3], Yi-Ping Hu[1, *], Min-Jun Wang[1, *]

© 2020. Zhu Y et al. Published online at http://www.aginganddisease.org/EN/10.14336/AD.2020.1227

SUPPLEMENTARY DATA

Supplementary table 1. Clinical characteristics of patients.

Patient number	Sex	Age	Type of disease	TB (mg/dL)	ALT (U/L)	AST (U/L)	AFP (ug/L)	HBV/HCV
1	Male	20	Hemangioma	0.57	10	17	2.1	N/N
2	Male	21	Hemangioma	0.31	8.1	10	1.5	N/N
3	Male	24	Hemangioma	0.39	11.6	21	1.7	N/N
4	Male	25	Hemangioma	0.81	7.8	11.5	1.3	N/N
5	Male	25	Hemangioma	0.45	16	18	1.9	N/N
6	Male	65	Hemangioma	1.03	19	26	1.6	N/N
7	Male	55	Hemangioma	0.78	10	14	2.5	N/N
8	Male	65	Hemangioma	0.91	8.1	8.9	1.7	N/N
9	Male	64	Hemangioma	0.57	7.8	11	2.4	N/N
10	Male	61	Hemangioma	0.86	9.4	8.7	2.8	N/N

TB: total bilirubin; ALT: alanine aminotransferase; AST: aspartate aminotransferase; AFP: alpha-fetoprotein; HBV: hepatitis B virus; HCV: hepatitis C virus; N: negative.

© 2020. Zhu Y et al. Published online at http://www.aginganddisease.org/EN/10.14336/AD.2020.1227

SUPPLEMENTARY DATA

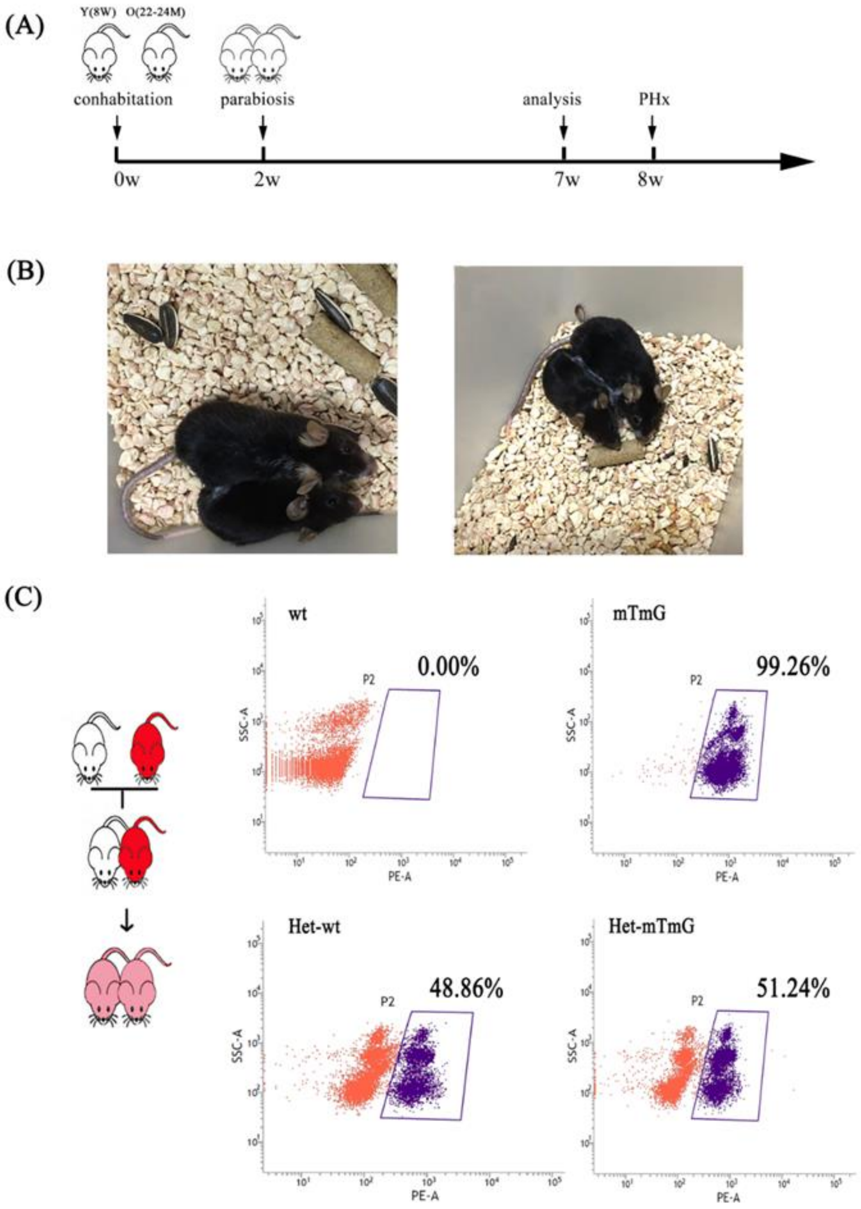

Supplementary Figure 1. Parabiosis model was established between young and aged mice. (A) The schematic diagram depicting parabiosis model and PHx in animals. W: Week; M: Month **(B)**The parabiotic partners live together in harmony. **(C)** Diagram depicting verification of the parabiosis model by flow cytometry analysis. Using parabiosis model established between Rosa26-mTmG^{flox} mice and wild-type mice. Rosa26-mTmG^{flox} mice was a global reporter mouse, allowing visualization of mTomato makers in fixed samples of all blood cells examined. tdTomato-positive blood cells were identified (~50%) in the peripheral blood of wild-type mice (Het-wt), and tdTomato-negative blood cells also represented in its' partner (Het-mTmG) after 2 weeks later (n=3 pairs).

© 2020. Zhu Y et al. Published online at http://www.aginganddisease.org/EN/10.14336/AD.2020.1227

SUPPLEMENTARY DATA

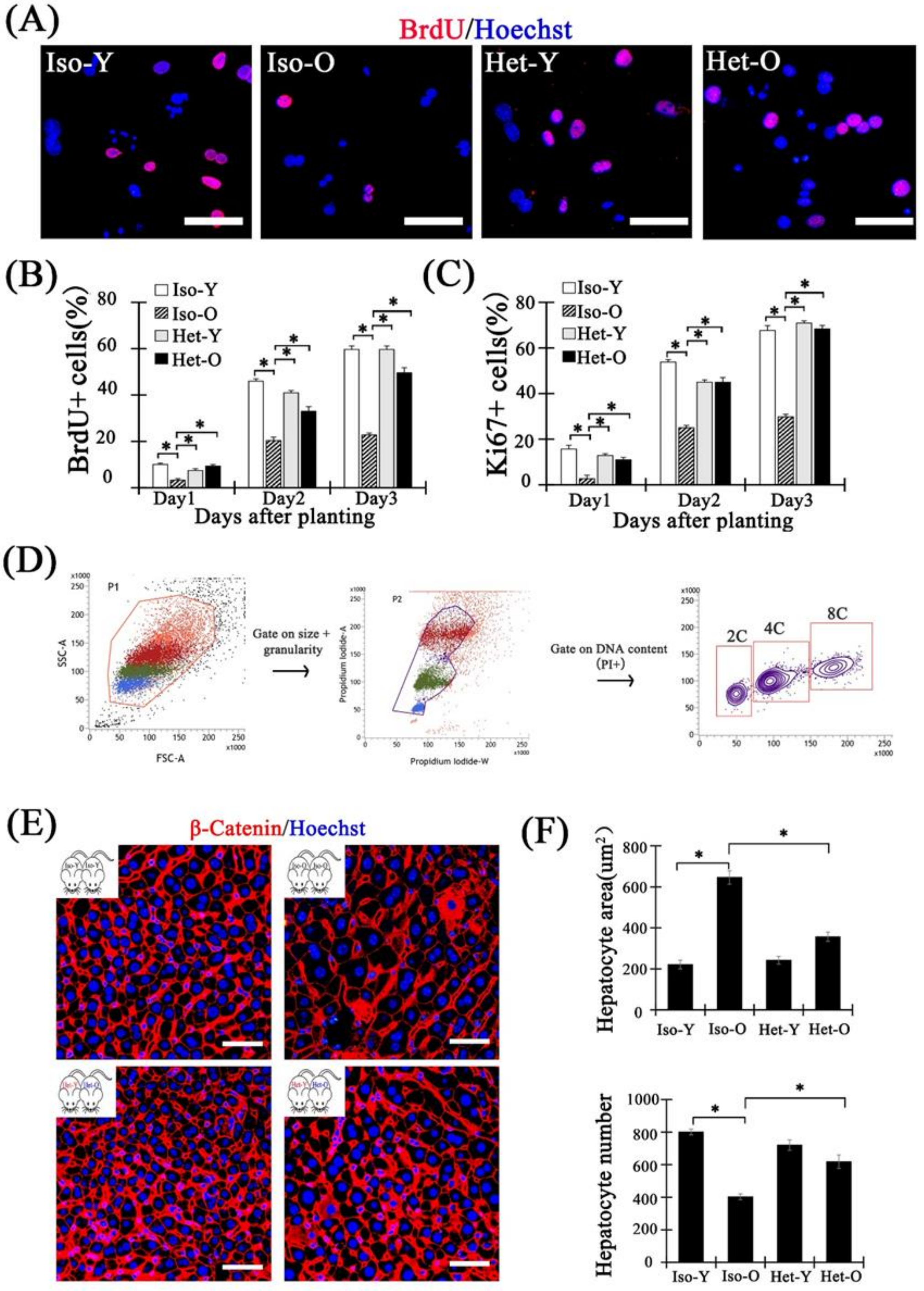

Supplementary Figure 2. The polyploidy reduction and autonomous proliferation in aged liver subjected to young milieu. (A) Immunofluorescence analysis of BrdU incorporation of primary hepatocytes isolated Iso-Y, Iso-O, Het-Y and Het-O mice at 3 days cultured *in vitro*. The bar=50μm, **(B-C)** The graph showed the percentage of BrdU-positive (B) and Ki67-positive (C) hepatocytes at day1, day2, and day3 during primary culture. Data are shown as mean ± SD. *p<0.05, n=5 pairs for each group. **(D)** Gating strategy for identifying hepatocyte populations based on DNA content. **(E)** Representative liver sections stained by Hoechst (nuclear labeling) and β-catenin (outline of hepatocytes) 5 weeks after parabiosis surgery (the bar=50μm). **(F)** Quantitative analysis the number and area of hepatocytes in 200-fold magnification for each group mice. Data are shown as mean ± SD. *p<0.05, n=5 pairs.

© 2020. Zhu Y et al. Published online at http://www.aginganddisease.org/EN/10.14336/AD.2020.1227

SUPPLEMENTARY DATA

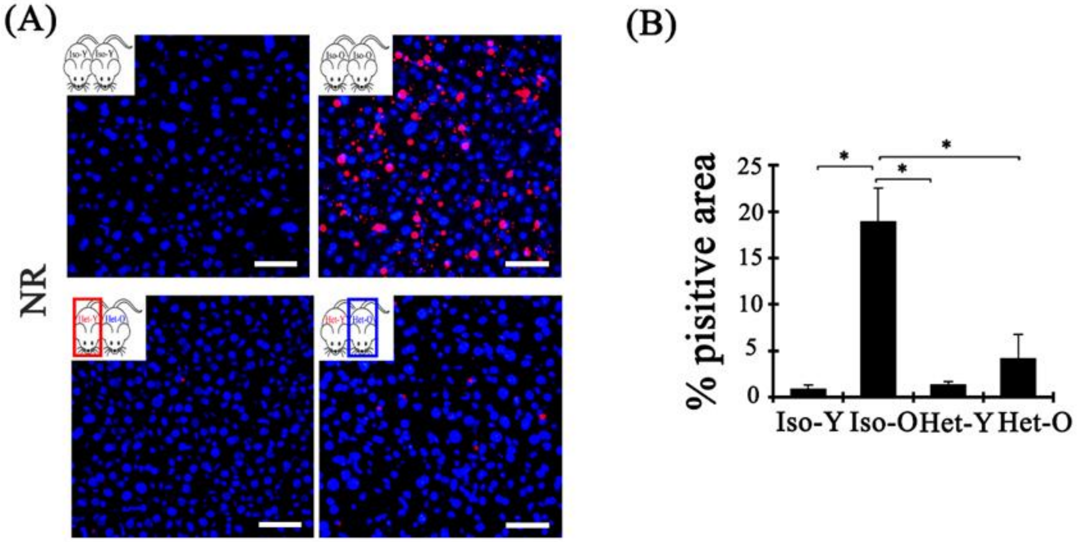

Supplementary Figure 3. The level of hepatic lipid accumulation in rodent liver. (A) Representative images showing Nile Red staining of frozen liver sections from respective group. The scale bars represent 50μm. **(B)** Percentage of Nile Red staining was determined using ImageJ. Data are shown as mean ± SD.*p<0.05, n=5 pairs.

© 2020. Zhu Y et al. Published online at http://www.aginganddisease.org/EN/10.14336/AD.2020.1227
